# Four-Year Outcomes of Anterior Pressed Lithium Disilicate Veneers Fabricated from 3D-Printed Burn-Out Patterns: A Clinical Case Report

**DOI:** 10.3390/dj14030175

**Published:** 2026-03-17

**Authors:** Suria Sarahi Oliver-Rivas, Carlos Roberto Luna-Domínguez, Rogelio Oliver-Parra, Ricardo De Jesus Figueroa-López, Gerardo Alberto Salvador Gomez Lara, Jorge Humberto Luna-Domínguez

**Affiliations:** Faculty of Dentistry, Autonomous University of Tamaulipas, Av. Universidad esq. con Blvd. Adolfo López Mateos s/n, Tampico C.P. 89337, Tamaulipas, Mexico; suria.oliver@uat.edu.mx (S.S.O.-R.); cldominguez@uat.edu.mx (C.R.L.-D.); roliver@docentes.uat.edu.mx (R.O.-P.); ricardo.figueroa@uat.edu.mx (R.D.J.F.-L.); gerardo.gomez@docentes.uat.edu.mx (G.A.S.G.L.)

**Keywords:** lithium disilicate veneers, 3D-printed burn-out pattern, additive-plus-pressing workflow, digital dentistry, anterior esthetics, CAD/CAM

## Abstract

**Background/Objectives:** Lithium disilicate (LD) veneers are widely used for minimally invasive anterior rehabilitation because of their favorable optical and mechanical properties. Fully digital workflows have been proposed as alternatives to conventional milling. These approaches combine computer-aided design and manufacturing (CAD/CAM) with 3D-printed burn-out patterns and subsequent heat pressing of LD ingots. However, clinical documentation of multi-unit anterior cases fabricated exclusively through this additive-plus-pressing route remains scarce. This case report aims to describe a fully digital additive-plus-pressing workflow for four maxillary anterior LD veneers and to report 48-month clinical outcomes. **Case Presentation:** A 52-year-old female presented with esthetic concerns involving the maxillary central and lateral incisors (teeth 11, 12, 21, and 22). After clinical and radiographic evaluation, a minimally invasive veneer-based rehabilitation was planned. Preparations were performed under magnification, and immediate dentin sealing was applied. Digital impressions were obtained with an intraoral scanner, and veneers were designed using CAD software(Exocad DentalDB 3.0 Galway (Exocad GmbH, Darmstadt, Germany). Castable resin patterns were 3D-printed, invested, and heat-pressed using LD ingots, followed by finishing and glazing. Adhesive cementation was performed under rubber dam isolation after hydrofluoric acid etching and silanization of the intaglio surfaces and conditioning of the tooth substrates according to the adhesive protocol, using a dual-cure resin cement. At the 48-month follow-up, all veneers remained intact, with clinically acceptable marginal adaptation, stable color and surface gloss, and no signs of secondary caries or marginal discoloration. The patient reported sustained esthetic satisfaction and comfortable function without postoperative sensitivity. **Conclusions:** This single-patient report suggests that a fully digital additive-plus-pressing workflow may be clinically viable for high-demand anterior LD veneers, providing favorable medium-term esthetics and patient-centered outcomes with no technical or biological complications. The reproducible protocol described may facilitate the integration of 3D printing and heat pressing into digital veneer rehabilitation and supports further controlled clinical investigations.

## 1. Introduction

Monolithic ceramic restorations have become widely used in dentistry for their ability to fulfill functional and esthetic requirements [1,2,3,4]. Among glass-based ceramics, lithium disilicate (LD) is regarded as a reference material. This is due to its high flexural strength, favorable optical properties, well-established bonding protocols, and long-term clinical performance [5,6,7]. Malament et al. reported 10-year survival rates above 98% for monolithic lithium disilicate restorations [8]. Similarly, Lindner et al. documented cumulative survival rates of 97.5% at approximately 6 years for pressed LD restorations, reinforcing the longevity of this material [9].

The adoption of digital workflows based on computer-aided design and manufacturing (CAD/CAM) has transformed restorative fabrication. These workflows improve efficiency and standardization compared with conventional techniques [10,11]. In most systems, LD restorations are fabricated through subtractive manufacturing (milling) of pre-crystallized ceramic blocks. This approach delivers high precision but is inherently limited by bur geometry and tool-path access. These limitations can affect preparation complexity, especially in ultra-thin areas and fine anatomical details [12,13,14].

As an alternative, hybrid workflows combining CAD with additive manufacturing have been proposed. In this “additive-plus-pressing” approach, a castable resin pattern is 3D-printed from the CAD design. This pattern is then invested and pressed to obtain the definitive ceramic restoration. In vitro studies showed that restorations produced with 3D-printed patterns achieved marginal and internal gaps within clinically acceptable thresholds. In some situations, they demonstrated equal or superior adaptation compared with conventional wax or milled patterns [15,16,17].

Despite these potential advantages, most available evidence on additive-plus-pressing workflows remains laboratory-based, and detailed clinical documentation of multi-unit anterior rehabilitations fabricated exclusively through this route is scarce. Emerging clinical data indicate comparable short-term performance between fully digital milled workflows and hybrid printed-pattern workflows [18], but medium-term clinical outcomes remain underreported. Therefore, this report presents 48-month outcomes of a multi-unit anterior veneer rehabilitation fabricated exclusively using a fully digital additive-plus-pressing workflow based on 3D-printed burn-out patterns and heat-pressed lithium disilicate. In addition to reporting clinical outcomes, we outline practical clinical and laboratory control points intended to support reproducibility and routine digital laboratory integration.

## 2. Case Presentation

**Figure 1:**
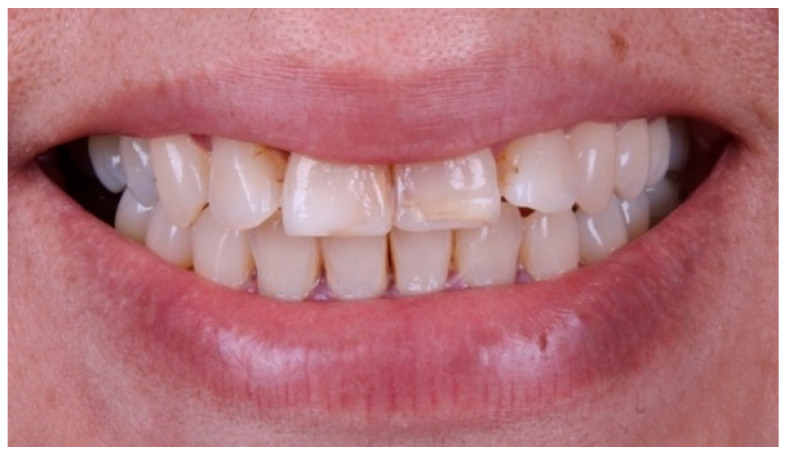
Baseline clinical photograph showing discoloration of the incisal edges of teeth 11 and 21 and slight labial protrusion of teeth 12 and 22.

A 52-year-old female patient with no relevant systemic conditions presented to the Prosthodontics Clinic at the Universidad Autónoma de Tamaulipas with a chief complaint of poor esthetics in her maxillary incisors. Intraoral examination revealed discoloration at the incisal edges of the maxillary central incisors (teeth 11 and 21) and slight labial protrusion of the maxillary lateral incisors (teeth 12 and 22) (Figure 1). There were no signs or symptoms of temporomandibular disorders or masticatory muscle tenderness. The patient’s general medical history was unremarkable, and no parafunctional habits or history of dental trauma were reported. Pulp sensibility testing (thermal cold test) elicited positive vitality responses within normal limits for all maxillary anterior teeth. Periodontal assessment revealed generalized gingival health with probing depths <3 mm and no bleeding on probing. Periapical radiographs showed normal bone levels and intact periodontal ligament spaces, with no apical pathology. Based on the clinical and radiographic findings, a minimally invasive restorative treatment plan involving lithium disilicate ceramic veneers on teeth 11, 12, 21, and 22 was proposed within a fully digital additive-plus-pressing workflow. The patient provided written informed consent for all clinical procedures, clinical photography, and case publication.

### 2.1. Tooth Preparation, Immediate Dentin Sealing, and Digital Impression

**Figure 2:**
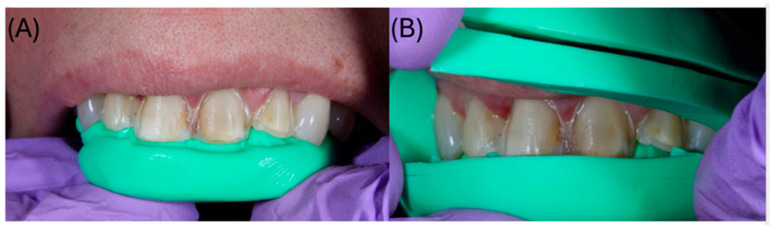
(**A**) Verification of incisal reduction using a silicone putty index; (**B**) verification of facial (vestibular) reduction using the same silicone guide (Silicon Edge Putty Soft, 900 g; MDC^®^).

**Figure 3:**
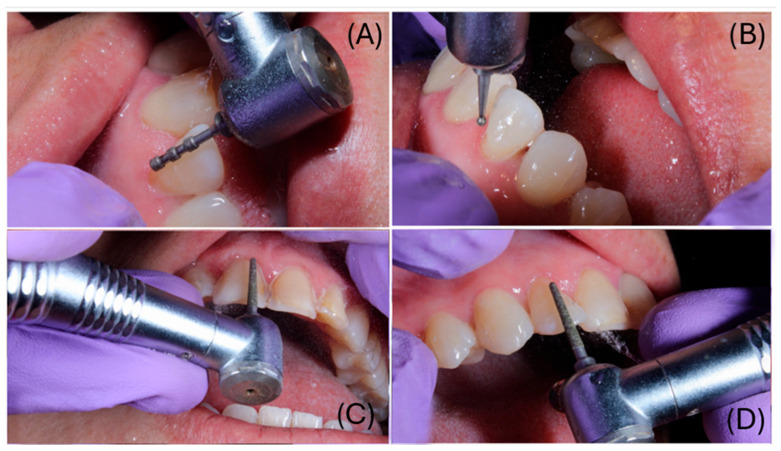
(**A**) Depth-limiting preparation in the cervical area to 0.6 mm; (**B**) standardization of facial reduction using 0.6 mm depth-orientation grooves; (**C**) removal of the depth grooves; (**D**) smoothing and regularization of the facial surface.

Under local anesthesia, veneer preparations were performed under magnification using a dental operating microscope (MAGNA, Labo America, Inc., Fremont, CA, USA), following minimally invasive principles. Approximately 0.6 mm of facial enamel reduction and 1.0 mm of incisal reduction were carried out. The preparation terminated in a circumferential 0.6 mm chamfer at the cervical margin (Figure 2). Depth-limiting diamond burs were used to standardize the reduction. Orientation grooves of 0.6 mm were prepared on the facial surface and subsequently blended to obtain a uniform reduction (Figure 3). Silicone putty indices (Silicon Edge Putty Soft, MDC Dental, Zapopan, Mexico) fabricated from the 3D-printed model of the digital diagnostic wax-up were used to verify reduction relative to the planned veneer thickness (Figure 2).

**Figure 4:**
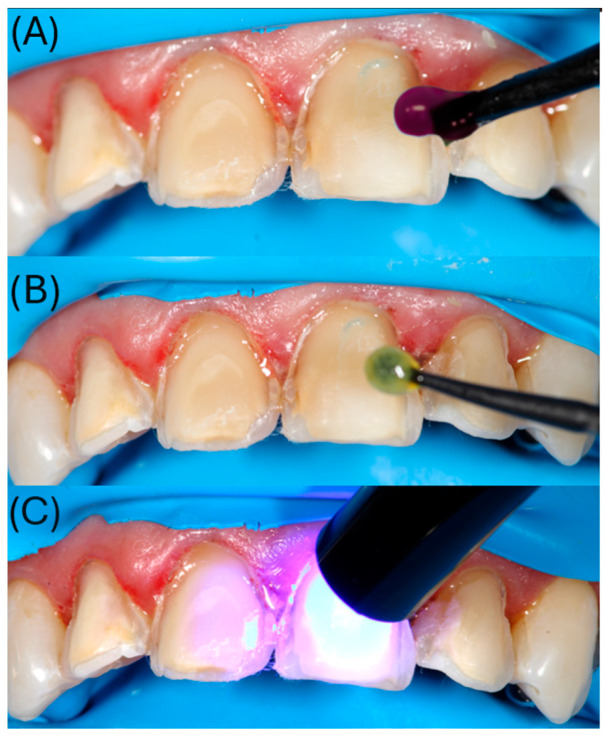
(**A**) Etching of enamel and dentin with 37.5% phosphoric acid gel (Kerr Gel Etchant; Kerr, Orange, CA, USA); (**B**) application of OptiBond FL primer (Kerr, Orange, CA, USA) with a microbrush and active rubbing for 15 s; (**C**) light curing with a Valo LED curing unit (Ultradent Products Inc., South Jordan, UT, USA).

After finishing the preparations, an immediate dentin sealing (IDS) protocol was performed using a three-step etch-and-rinse adhesive system (OptiBond FL, Kerr, Orange, CA, USA) (Figure 4). Enamel and dentin were etched with 37.5% phosphoric acid gel for 15 s, rinsed, and gently air-dried. The primer was applied to dentin with a microbrush for 15 s and light-cured. This was followed by the application of the adhesive resin and polymerization according to the manufacturer’s instructions (Figure 4).

**Figure 5:**
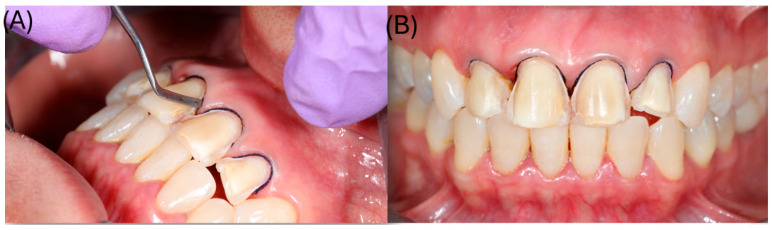
(**A**) Placement of #000 retraction cord (Ultrapak; Ultradent Products Inc., South Jordan, UT, USA); (**B**) frontal view of the prepared teeth with the retraction cord in place.

**Figure 6:**
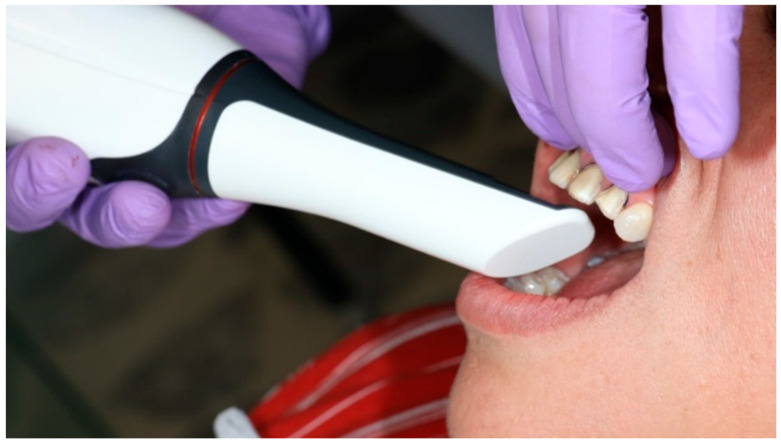
Digital impression of teeth 11, 12, 21, and 22 obtained with a TRIOS^®^ intraoral scanner (3Shape A/S, Copenhagen, Denmark).

Subsequently, a #000 retraction cord (Ultrapak^®^, Ultradent Products Inc., South Jordan, UT, USA) was placed in the gingival sulcus around each prepared tooth to achieve circumferential gingival displacement and clear exposure of the finishing lines prior to digital impression (Figure 5). The maxillary and mandibular arches were then scanned with an intraoral scanner (TRIOS^®^, 3Shape A/S, Copenhagen, Denmark) immediately after preparation, ensuring that the tooth surfaces were clean and dry. The scanning protocol consisted of recording the occlusal surfaces first, followed by the facial and palatal aspects, generating a precise three-dimensional virtual model of the prepared teeth and their occlusal relationship (Figure 6).

**Figure 7:**
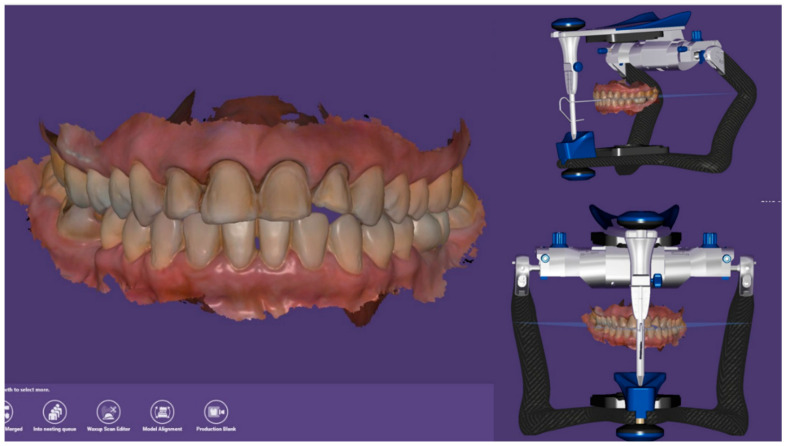
Mounting of the digital casts on a virtual articulator in Exocad DentalDB 3.0 Galway (Exocad GmbH, Darmstadt, Germany).

**Figure 8:**
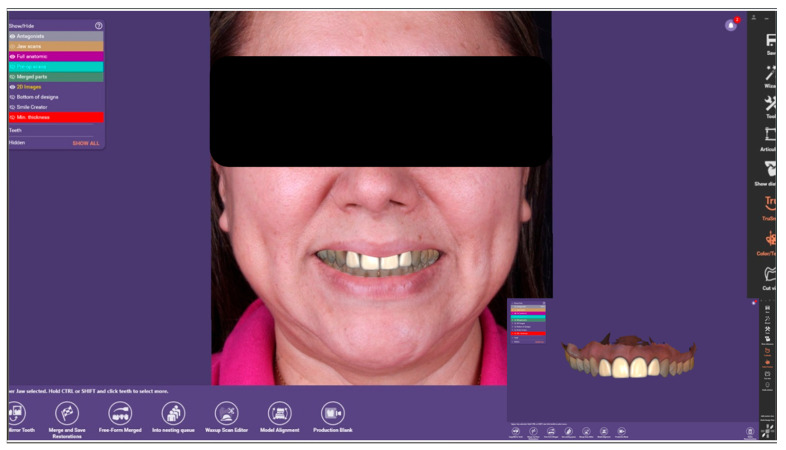
Final CAD design of the veneers for teeth 11, 12, 21, and 22 in Exocad DentalDB 3.0 Galway (Exocad GmbH, Darmstadt, Germany).

Using the scan data, the definitive veneers were designed with CAD software (Exocad DentalDB 3.0 Galway, Exocad GmbH, Darmstadt, Germany). The virtual casts were mounted on a digital articulator to evaluate static and dynamic occlusion (Figure 7), and individual tooth morphology was refined until the desired shape, proportions, and incisal guidance were achieved for teeth 11, 12, 21, and 22 (Figure 8). Between appointments, provisional restorations were fabricated using a bis-acrylic resin (LuxaCrown^®^, DMG, Hamburg, Germany) and a silicone index derived from the diagnostic wax-up, providing adequate function and esthetics during laboratory fabrication of the definitive veneers.

### 2.2. Additive Pattern Fabrication and Heat-Pressing of Lithium Disilicate Veneers

**Figure 9:**
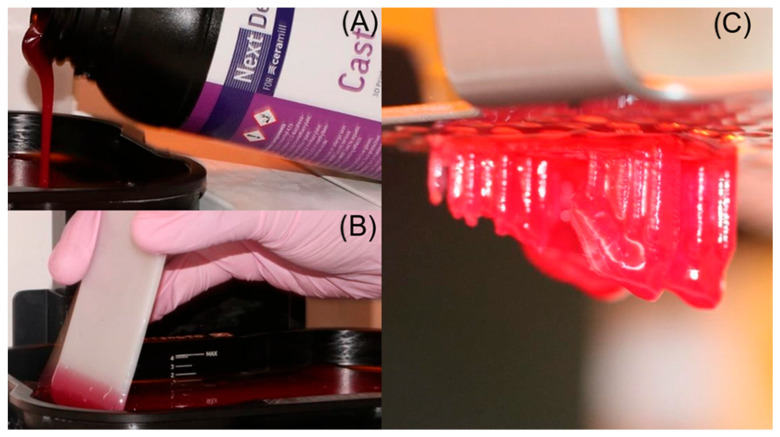
(**A**) 3D-printed castable resin patterns on the build platform of the 3D printer NextDent 5100; (**B**) post-print inspection and cleaning of the print environment; (**C**) veneer patterns corresponding to teeth 11, 12, 21, and 22 printed in castable resin.

The digital veneer designs were exported to a Digital Light Processing (DLP) 3D printer (NextDent^®^ 5100, NextDent B.V., Soesterberg, The Netherlands) for the fabrication of burn-out resin patterns. A castable resin specifically formulated for investment and burn-out was used (NextDent^®^ Cast, NextDent B.V., Soesterberg, The Netherlands). After printing, the patterns were cleaned in isopropyl alcohol and post-polymerized in a UV-light curing unit (LC-3DPrint Box, NextDent B.V., Soesterberg, The Netherlands) for 5 min, strictly following the manufacturer’s recommendations for castable materials (Figure 9).

**Figure 10:**
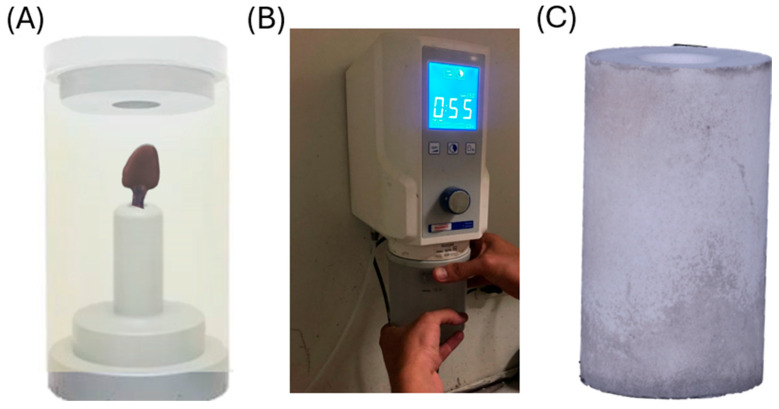
(**A**) 3D-printed castable resin patterns mounted on a sprue base and ready for investment; (**B**) preparation of phosphate-bonded investment (S&S SpeedVest; Scheftner Dental Alloys, Mainz, Germany) using a vacuum mixer; (**C**) investment ring filled with the mixed investment and the injection cup coated and ready for the burn-out cycle.

The printed patterns were invested using a high-temperature, phosphate-bonded investment material (S&S SpeedVest^®^, Scheftner Dental Alloys, Mainz, Germany). The investment was mixed under vacuum and poured into the ring (Figure 10). After setting, the rings were transferred to a preheating furnace for the burn-out cycle.

**Figure 11:**
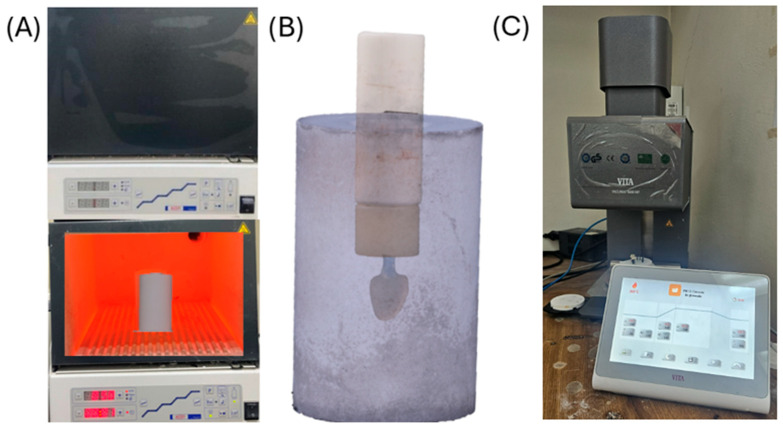
(**A**) Investment ring and injection cup placed in a preheating furnace (Magma; Renfert, GmbH, Hilzingen, Germany); (**B**) investment ring positioned in the pressing unit and ready for injection; (**C**) injection/pressing furnace (Vacumat 6000 MP; VITA, Zahnfabrik, Bad Säckingen, Germany) used to press lithium disilicate ingots (Amber^®^ Press; HASS Bio, Gangneung, Republic of Korea).

Burn-out and pressing were performed following the manufacturer’s recommended schedule for the castable resin and lithium disilicate ingots. Immediately after burn-out, the hot rings were placed in a pressing furnace (Vacumat^®^ 6000 MP, VITA Zahnfabrik, Bad Säckingen, Germany). Pressable lithium disilicate ingots (Amber^®^ Press, HASS Bio, Gangneung, Republic of Korea) in shade A2 were pressed following the recommended parameters. The molten ceramic filled the mold, reproducing the veneer morphology (Figure 11). After cooling, the investment was divested to retrieve the veneers.

### 2.3. Finishing and Glazing

**Figure 12:**
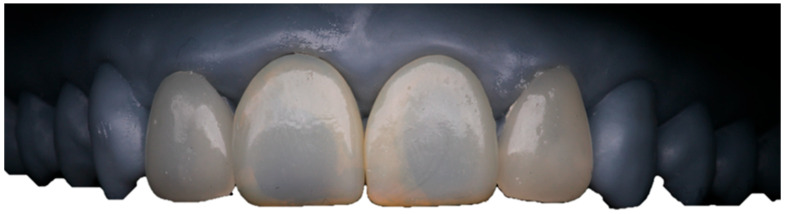
Final lithium disilicate veneers after contouring, mechanical polishing, and glazing, seated on the 3D-printed model to verify fit and proximal contacts before clinical cementation.

Residual investment material was removed by airborne particle abrasion using 50 µm aluminum oxide (Al_2_O_3_) at a pressure of 2 bar (0.2 MPa), strictly adhering to the manufacturer’s recommendations to preserve marginal integrity. The veneers were then cleaned and refined with fine diamond burs. Mechanical prepolishing was performed using silicone rubber polishers to smooth the surface texture. Subsequently, a glaze layer (VITA AKZENT^®^ Plus GLAZE LT, VITA Zahnfabrik, Bad Säckingen, Germany) was applied and fired under vacuum at 770 °C for 1 min to obtain a smooth, glossy surface and support esthetic stability. The finished veneers were seated on a 3D-printed model to verify fit and proximal contacts prior to clinical cementation (Figure 12).

### 2.4. Veneer Try-In and Adhesive Cementation

At the delivery appointment, the provisional restorations were removed. Each definitive veneer was tried in individually to verify marginal adaptation, cervical and proximal extension, and the planned shape. After passive seating and appropriate integration were confirmed and the patient approved the esthetic outcome, the veneers were removed, and adhesive cementation was performed under rubber dam isolation.

After try-in, the ceramic intaglio surfaces were cleaned and conditioned strictly following the protocol: 9% hydrofluoric acid etching for 20 s, rinsing, cleaning with 35% phosphoric acid for 15 s, ultrasonic cleaning in isopropyl alcohol for 3 min, drying, and silane application for 60 s.

**Figure 13:**
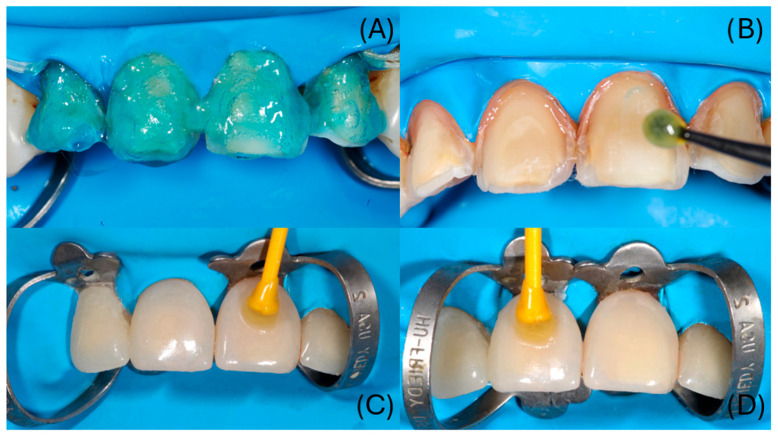
(**A**) Etching of enamel and dentin with 37.5% phosphoric acid gel (Kerr Gel Etchant; Kerr, Orange, CA, USA) for 15 s; (**B**) active application of OptiBond FL primer (Kerr, Orange, CA, USA) on dentin with a microbrush; (**C**,**D**) adhesive cementation of veneers on teeth 11, 12, 21, and 22 using dual-cure resin cement (Panavia V5; Kuraray Noritake Dental Inc., Kurashiki, Okayama, Japan).

Adjacent teeth were protected with polytetrafluoroethylene (PTFE) tape. Enamel and exposed dentin were etched with 37.5% phosphoric acid for 15 s, rinsed, and gently air-dried. The immediate dentin sealing (IDS) layer was reactivated using a three-step etch-and-rinse adhesive system (OptiBond FL, Kerr, Orange, CA, USA); primer actively applied for 15 s and air-thinned, followed by adhesive resin light-curing for 10 s) (Figure 13A,B). Dual-cure resin cement (Panavia V5, Kuraray Noritake Dental Inc., Kurashiki, Okayama, Japan); Translucent shade) was applied to the intaglio surfaces, and the veneers were seated with gentle pressure. Following a tack-cure to facilitate excess cement removal, glycerin gel was applied to minimize the oxygen inhibition layer, and each veneer was light-cured for 40 s from the facial and palatal aspects (Figure 13C,D).

**Figure 14:**
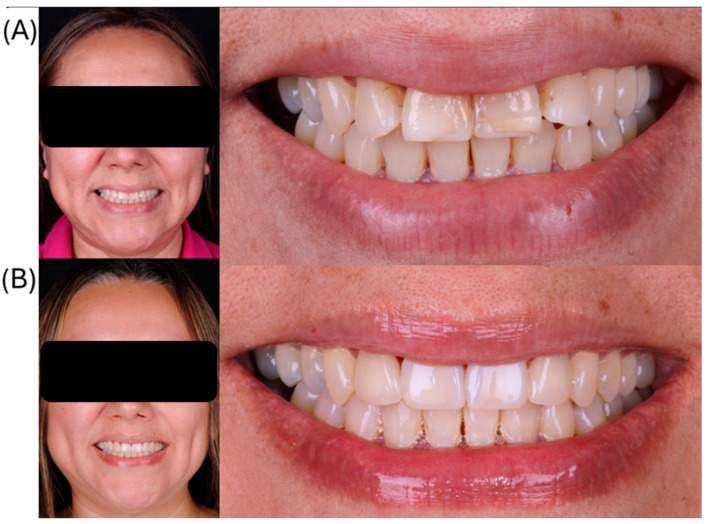
(**A**) Baseline intraoral and extraoral views of the maxillary anterior segment prior to treatment; (**B**) intraoral and extraoral views after placement of lithium disilicate veneers, showing improved esthetics and integration with the surrounding dentition.

Veneers were bonded sequentially using the same protocol. After rubber dam removal, occlusion was checked in maximum intercuspation and excursive movements, and minor premature contacts were adjusted and repolished. Shade integration was verified using a digital spectrophotometer (VITA Easyshade® V, VITA Zahnfabrik, Bad Säckingen, Germany), confirming shade A2. The immediate postoperative examination showed clinically acceptable marginal adaptation and optimal esthetic integration (Figure 14). The patient received detailed postoperative instructions, emphasizing the use of a soft-bristle toothbrush, interproximal flossing protocols, and dietary recommendations to avoid biting hard objects directly with the veneers. A biannual maintenance schedule was established to monitor periodontal health and restoration integrity.

## 3. Results

Clinical outcomes were assessed at delivery (baseline) and at the 48-month recall using modified USPHS/Ryge criteria based on clinical inspection, gentle tactile exploration, periapical radiographs, patient report, and instrumental shade verification (Table 1). At delivery, all four lithium disilicate veneers (teeth 11, 12, 21, and 22) seated passively without resistance and showed clinically acceptable marginal adaptation rated as Alpha. No visible overhangs or marginal gaps were detected on inspection, and follow-up periapical radiographs revealed no interfacial discrepancies between the veneers and the underlying tooth structure.

Over the 48-month follow-up period, no technical complications were recorded. There were no incidents of veneer fracture, incisal chipping, or debonding (Retention: Alpha). Esthetic outcomes remained stable; instrumental verification using a digital spectrophotometer (VITA Easyshade^®^ V, VITA Zahnfabrik, Bad Säckingen, Germany) at the 48-month recall confirmed the maintenance of the baseline shade (A2). Consequently, no perceptible superficial staining or marginal discoloration was observed clinically (Color match: Alpha), and the glazed surfaces maintained a smooth, glossy appearance. No abnormal wear facets or increased roughness were observed on the veneers, and no adverse effects on the opposing dentition were noted. No secondary caries was detected clinically or radiographically at the veneer margins.

Regarding biological and patient-reported outcomes, the periodontal tissues around teeth 11, 12, 21, and 22 remained healthy, with no bleeding on probing and no signs of inflammation or gingival recession. The patient reported comfortable function during biting and mastication, absence of postoperative sensitivity, and ease of daily oral hygiene. At the 48-month recall, she remained satisfied with the esthetic outcome and overall appearance of her smile.

## 4. Discussion

This case report describes a fully digital workflow in which laminate veneers were designed using computer-aided design, fabricated from 3D-printed castable resin burn-out patterns, and completed by heat pressing lithium disilicate. The rehabilitation involved four maxillary anterior veneers. At the 48-month follow-up, the restorations remained clinically stable with clinically acceptable marginal adaptation, stable esthetics, sustained patient satisfaction, and no technical or biological complications. By providing medium-term follow-up of a multi-unit anterior rehabilitation fabricated exclusively through this workflow, the present report extends the currently available clinical evidence beyond predominantly laboratory investigations. These findings are consistent with long-term clinical evidence supporting lithium disilicate veneers and other partial-coverage restorations, where 10-year survival rates of approximately 96–98% have been reported [5,6,7,8,9].

Digital workflows that combine intraoral scanning, virtual planning, and CAD/CAM manufacturing are increasingly used for veneer fabrication. Clinical reports and retrospective series have reported high survival and success rates for lithium disilicate veneers, together with acceptable marginal outcomes and favorable patient-reported esthetic results [10,11,19,20]. Most available clinical evidence, however, still focuses on subtractive manufacturing from ceramic blocks. While milling generally produces restorations within clinically acceptable adaptation thresholds, the process is constrained by bur geometry and toolpath access, which can limit reproduction of very thin areas and fine anatomic details in selected situations [12,13,14].

Laboratory studies comparing pressed lithium disilicate restorations fabricated from conventional wax patterns, milled wax, or 3D-printed castable resin patterns have generally reported marginal and internal gaps within clinically acceptable limits, often without clinically meaningful differences among pattern fabrication methods [15,16,17,21]. In addition, a randomized clinical trial suggested comparable short-term clinical performance between milled workflows and printed-pattern plus pressing workflows [18]. Together, these findings indicate that incorporating a digital pattern step into a pressed ceramic workflow is unlikely to compromise adaptation when the protocol is carefully executed. In the present case, the passive seating observed clinically suggests that the dimensional accuracy of the 3D-printed patterns was successfully maintained. This is likely attributable to the strict adherence to the manufacturer’s post-polymerization parameters (specific light unit and time) and the use of a matched investment material, which are critical factors for controlling expansion during burn-out. Nonetheless, the present report did not include quantitative metrology of marginal or internal fit, so the clinical observations should be interpreted accordingly.

From a prosthodontic design perspective, printing the burn-out pattern may provide practical advantages in the anterior segment. Additive pattern fabrication can allow accurate reproduction of delicate incisal morphology, subtle surface texture, and transition lines that may be difficult to reproduce by milling because of bur diameter and toolpath limitations [12,15,16,17]. The digital design can also be archived and duplicated if remakes are required, which may improve reproducibility and clinician-laboratory communication. This indirect, pattern-based strategy differs from direct 3D printing of lithium disilicate veneers, such as lithography-based ceramic manufacturing, which has shown promising feasibility in laboratory studies but still has limited clinical evidence and requires specialized equipment and sintering protocols [22,23].

Optical integration is a key objective in anterior veneer therapy. Lithium disilicate offers favorable translucency and can be individualized through finishing and glazing, which may help maintain surface gloss and reduce extrinsic staining over time. In this case, shade and translucency were clinically stable, and no marginal discoloration was observed at 48 months. This preservation of marginal integrity was likely facilitated by the controlled devesting protocol using low-pressure airborne-particle abrasion (2 bar), which minimizes the risk of micro-chipping the thin ceramic margins. The outcome is also influenced by the adhesive strategy. Long-term success of lithium disilicate veneers depends on careful conditioning of the ceramic surface, appropriate management of the tooth substrate, and resin cementation under strict isolation [5,6,7,19]. Notably, the cleaning protocol employed (hydrofluoric acid followed by phosphoric acid and ultrasonic cleaning) aimed to eliminate insoluble salts, contributing to the durable bond. Furthermore, the protocol incorporated immediate dentin sealing (IDS). The absence of postoperative sensitivity recorded at follow-up supports the efficacy of IDS in protecting the dentin-pulp complex and optimizing the bonding interface [19,20,21,22,23].

Several limitations should be acknowledged. This is a single-patient case report without a comparison group, so generalizability is limited and the findings cannot be used for statistical inference. No quantitative assessment of marginal gap, internal fit, or optical stability was performed; techniques such as replica methods, micro-computed tomography, high-resolution microscopy, or spectrophotometric color analysis would be necessary to compare this case with laboratory benchmarks [15,16,17,21]. In addition, the 48-month follow-up represents a medium-term interval relative to decade-long series available for lithium disilicate veneers [5,6,7,8,9]. Implementation factors, including access to a reliable 3D printer, pressing equipment, and trained personnel, may influence cost and adoption. Future research should include prospective clinical studies and randomized trials comparing milled, pressed, and additive-based workflows using standardized measures of adaptation, esthetic stability, mechanical performance, and patient-reported outcomes.

## 5. Conclusions

This case report demonstrates that a fully digital additive-plus-pressing workflow—combining intraoral scanning, CAD design, 3D-printed burn-out patterns, and heat-pressed lithium disilicate—represents a viable and effective treatment modality for high-demand anterior rehabilitation. By overcoming the geometric limitations of subtractive manufacturing, this protocol allowed for the precise reproduction of intricate esthetic details while maintaining the established mechanical reliability of pressed ceramics.

In this patient, the strict adherence to calibrated manufacturing and adhesive protocols (including immediate dentin sealing) resulted in restorations with passive seating, optimal marginal adaptation, and durable esthetic integration at the 48-month follow-up, with no recorded technical or biological complications.

These outcomes should be interpreted within the limitations of a single-patient case report without a comparison group. Further prospective studies and controlled clinical trials are needed to compare additive-plus-pressing workflows with milled and conventional pressed approaches using standardized metrology and patient-reported measures. From a clinical perspective, this workflow offers a practical advantage by integrating digital efficiency with the predictability of pressed lithium disilicate, without requiring the specialized equipment needed for direct ceramic printing.

## Figures and Tables

**Table 1:** Clinical outcomes at delivery and at the 48-month follow-up assessed using modified USPHS/Ryge criteria for four maxillary lithium disilicate veneers (teeth 11, 12, 21, and 22).

Criterion (Modified USPHS/Ryge)	Delivery (Baseline)	48-Month Follow-Up	Clinical Basis (as Documented in This Report)
Color match	Alpha	Alpha	Shade match (A2) achieved at delivery; instrumental verification (Easyshade V) confirmed stability (A2) at 48 months.
Marginal discoloration	Alpha	Alpha	No marginal staining/discoloration observed clinically during follow-up.
Surface texture/luster	Alpha	Alpha	Glazed surfaces at delivery; smooth and glossy appearance maintained clinically at 48 months.
Marginal integrity/adaptation	Alpha	Alpha	Clinically acceptable marginal adaptation with no detectable overhangs or gaps; radiographs showed no interfacial discrepancies.
Anatomic form/contour	Alpha	Alpha	No clinically relevant overcontouring noted; patient reported easy hygiene and no plaque-retentive areas related to veneer contours.
Retention	Alpha	Alpha	No debonding events recorded over 48 months.
Fracture/chipping	Alpha	Alpha	No veneer fractures or incisal chipping recorded during follow-up.
Secondary caries	Alpha	Alpha	No secondary caries detected clinically or radiographically during follow-up.

Modified USPHS/Ryge ratings: Alpha = clinically ideal; Bravo = minor deviation but clinically acceptable; Charlie = clinically unacceptable (repair indicated); Delta = failure (replacement indicated). Ratings were based on clinical inspection, gentle tactile exploration, periapical radiographs, patient report, and spectrophotometric verification. Quantitative metrology of marginal/internal fit (e.g., micro-CT) was not performed.

## Data Availability

The data presented in this study are available on request from the corresponding author.
